# HAWKFOG-an enhanced deep learning framework for the Fog-IoT environment

**DOI:** 10.3389/frai.2024.1354742

**Published:** 2024-06-28

**Authors:** R. Abirami, Poovammal E

**Affiliations:** Department of Computing Technologies, SRM Institute of Science and Technology, Kattankulathur, Chennai, India

**Keywords:** cardiac diseases, internet of things, Harris hawk optimization, fog/edge computing, self attention mechanism

## Abstract

Cardiac disease is considered as the one of the deadliest diseases that constantly increases the globe’s mortality rate. Since a lot of expertise is required for an accurate prediction of heart disease, designing an intelligent predictive system for cardiac diseases remains to be complex and tricky. Internet of Things based health regulation systems are a relatively recent technology. In addition, novel Edge and Fog device concepts are presented to advance prediction results. However, the main problem with the current systems is that they are unable to meet the demands of effective diagnosis systems due to their poor prediction capabilities. To overcome this problem, this research proposes a novel framework called HAWKFOGS which innovatively integrates the deep learning for a practical diagnosis of cardiac problems using edge and fog computing devices. The current datasets were gathered from different subjects using IoT devices interfaced with the electrocardiography and blood pressure sensors. The data are then predicted as normal and abnormal using the Logistic Chaos based Harris Hawk Optimized Enhanced Gated Recurrent Neural Networks. The ablation experiments are carried out using IoT nodes interfaced with medical sensors and fog gateways based on Embedded Jetson Nano devices. The suggested algorithm’s performance is measured. Additionally, Model Building Time is computed to validate the suggested model’s response. Compared to the other algorithms, the suggested model yielded the best results in terms of accuracy (99.7%), precision (99.65%), recall (99.7%), specificity (99.7%). F1-score (99.69%) and used the least amount of Model Building Time (1.16 s) to predict cardiac diseases.

## 1 Introduction

In accordance with the World Health Organization (WHO) 2020 study, an estimation of 17.9 million individuals die from cardiac disease every year, making it the primary cause of mortality in the world. According to the Global Burden of Disease (GBD) research the death rate from cardiac illness is 272 per 100,000 persons throughout all of India, along with the poorer regions and rural areas, which is significantly greater than the global mean of 235 (Gill et al., 2018). The number of death rate due to cardiac disease is increasing exponentially day by day. Early-stage detection of Cardiac Disease and receiving appropriate treatment for preventing premature deaths is an important way of reducing this toll. So, diagnosis of cardiac disease requires being more accurate, precise, and reliable approach. Since a lot of expertise is required for an accurate, precise and reliable prediction of cardiac disease, designing an intelligent predictive system for cardiac diseases remains to be complex and tricky. Newer technologies such as “fog computing, Internet of Things (IoT), and edge computing” have experienced substantial growth because they can offer a range of reaction qualities depending on the applications for which they are intended (He et al., 2017). A wide range of algorithms can be used with IoT- based health monitoring systems, a relatively new technology that enables precise disease classification from sensor data. In addition, novel Edge and Fog computing concepts are presented to advance prediction performances. In recent year, IoT together with Edge and data may be quickly and effectively analyzed in real-time with fog computing. Because of these technologies, applications that require low latency are realistic can benefit the most from fog computing in regards to portability, privacy, secure, low latency, as well as network bandwidth (Abdullahi et al., 2015; Satyanarayanan, 2017; Gill et al., 2019; Goyal et al., 2019; Mutlag et al., 2019).

One of the key areas of application that needs accurate and reliable results is the detection of heart disease, and fog computing has been applied in this area to portray the process in a brighter light. Resources are provided nearer to users through fog computing, which reduces latency and lengthens the lifespan of patients. But getting the outcome is not enough as with such complex data as we cannot sacrifice the diagnosis accuracy. Deep learning algorithms have grown rapidly in recent times across a different kind of industries, including healthcare, industrial automation, even processing natural language. Recent models using deep learning for health care data applications, however, are extremely complex and demand a significant computing resource for both training and prediction (Lim et al., 2000). These intricate neural networks require a lot of training time before they can be used for data analysis. The more complex the network, the greater the forecast time, and the higher the accuracy needed (Datta and Sharma, 2017). This has been a significant issue regarding IoT applications in the healthcare industry and other fields where real-time data are essential. Given that computing on the edge or in the fog has the significant benefit of speeding up response times, this opens up a new line of inquiry into the integration of resource-conscious, complicated deep learning models.

To provide high detection performance, low latency, low resource utilization, and health care to individuals with heart issues and other users requiring real-time performance, an integrated Fog/Edge based model has to be implemented. For the Fog devices to meet the aforementioned requirements, several deep learning algorithms (Acharya et al., 2017; Ali and Ghazal, 2017; Azimi et al., 2018; Buch et al., 2018; Panch et al., 2019; Juarez-Orozco et al., 2020) are developed and tested. Yet not every deep learning framework on the market is suitable for a deployment that delivers high accuracy and quick response times. This study was motivated by the lack of existing frameworks that meet the aforementioned requirements. Recently, integration of meta-heuristic algorithms over the deep learning models has gained the better light of research to obtain the stable network that can aid for better diagnosis and clinical treatments. These algorithms also plays a pivotal role in selecting and tuning the hyperparameters that steers the DL models. In spite of several meta-heuristic algorithms, achieving the stable networks configurable for IoT-Edge networks still remains to be real challenge.

In this study, an intelligent healthcare system based on fog for the monitoring of cardiac heart disease is Hawk Fogs using the novel attention gated recurrent neural networks integrated with the logistic chaos based Harris hawk tuned feed forward learning networks. Hawk Fogs provides cardiac disease monitoring and detection using the novel learning networks which are deployed in the Fog/Edge Devices. To the best of our knowledge, this is the first instance of optimised learning models being implemented in fog devices without compromising performance or efficiency. The primary contribution of the paper is as follows:

1. Developed the Low Complexity Attention Gated Recurrent Units integrated with the Feed Forward learning Networks for a suitable deployment in Fog devices.
2. Proposed the novel Chaos based Harris Hawk Optimization to tune the Hyper parameters of the feed forward learning networks for consuming the lesser model building time in which reduces the latency in predicting the heart diseases.
3. Generated the Experimental test beds for the real time data collection systems using IoT sensors boards and Fog devices using the Embedded Jetson Nano boards.
4. Demonstrated and evaluated the suggested model’s functionality on built-in test beds. The suggested model suppresses the other models, and the deep learning model’s performance has been compared with that of other advanced deep learning models.

The essay is organized as follows for the remaining sections: The background information on various works by multiple authors is reviewed in Section 2. The suggested hybrid deep learning model is presented in Section 3 together with the data collection unit and pre-processed data. The experimental setup and dataset details for the suggested model are presented in Section 4. The performance evaluation, ablation study, model building time analysis, experimental outcome analysis, and statistical outcome validation method are presented in Section 5 after Section 4. Section 6 concludes the analysis by outlining its future scope.

## 2 Background works

Jenifer et al. (2022) proposal seeks to use the Internet of Things to create an edge-based heart illness predicting device. An accelerometer, temperature, and pulse rate sensor were all linked to a Raspberry Pi, which was used to create the prototype. In order to interpret the patient’s condition, a trained machine learning algorithm is employed to evaluate the measured value. Also, the system was put to the test in a variety of situations, and it performed 88% accurately. This framework offers the benefit of a high learning rate. Unfortunately, it is unsuitable for a real-time situation (Jenifer et al., 2022).

In an effort to create a smart healthcare paradigm, Raju et al. (2022) used edge fog and cloud computing. This model relied on an array of pieces of hardware to collect its information. Amplitude, overall harmonic distortion, standard deviation, zero crossing rate, pulse rate, entropy, and energy were all calculated in order to extract the cardiac characteristics. Using the Cascaded Convolutional Neural Network (CCNN) and the Galactic Swarm Optimization (GSO) method to optimize certain CNN metrics, all these characteristics were included to the diagnostic system. Accuracy, specificity, sensitivity, precision, and F1-score performance are all improved by this approach. Although it may be utilized in a real-time setting, it does not increase forecast accuracy (Raju et al., 2022).

Edge computing and IoT concepts were used by Gupta et al. (2023) to create a CNN-based prediction model. A distributed environment architecture called edge computing allows regional edge servers that have been estimated at the endpoint of IoT devices, it enables speedy resource availability and reaction times. The health data that IoT devices collect is analyzed using the CNN model. The purpose of edge devices is also to deliver timely health forecast reports to patients and physicians via edge servers. Performance metrics for accuracy and error rate analysis of the suggested mechanism are available. A superior accuracy of 99.23% was attained by this framework. Nonetheless, this framework’s greater energy usage has been noted as a disadvantage (Gupta et al., 2023).

A Deep Learning Modified Neural Network (DLMNN)-based framework for diagnosing cardiac disease was created by Sarmah (2020). To start, the correct hospital is authenticated using SHA-512. Despite being linked to the sick person’s body, the wearable IoT sensor device simultaneously transmits sensor information to the cloud. Before any sensor data is sent to the cloud, it is first safely encrypted using the AES algorithm. The DLMNN classifier is used to finish the classification after the encrypted data has been decrypted. This framework’s key benefit is its high level of security, but a significant disadvantage is that optimization techniques, which demand additional training time, are not included (Sarmah, 2020).

The Cascaded Long Short Term Memory (CSO-CLSTM) framework for illness diagnostics was introduced by Mansour et al. (2021). CSO is employed for fine-tune both the “weights” and to improve the medical data’s categorization, “bias” factors of the CLSTM model were used. In this research, the outliers are also removed using the isolation Forest (iForest) approach. The results of the diagnostic tests using the CLSTM model are significantly improved by the application of CSO. During testing, the CSO-LSTM model given was able to diagnose diabetes and heart disease with maximum accuracy of 97.26 and 96.16%, respectively. Nevertheless, it does not take security concerns into account and uses less energy (Mansour et al., 2021).

To improve cardiac arrhythmia analysis and acute stroke prediction’s precision and speed, Lavanya et al. (2023) introduced an edge computing technique. To identify cardiac arrhythmia and anticipate acute stroke, a CNN model based on deep learning is created. The deep learning model is trained using real-time data from the MIT-BIH as well as physiological data and heart rate characteristics. More accurately than many other feature extraction and classification techniques now in use are the DWT based feature extraction as well as deep CNN based multiclass classification. This classifier achieves 99.4% sensitivity, 99.1% specificity, and 99.3% accuracy. Yet this framework’s primary flaw is its lack of compatibility (Lavanya et al., 2023).

By using machine-learning techniques like fuzzy c-means neural networks (FNN) and a deep convolution NN, Venkatesan et al. (2022) hope to recognize the crucial cardiac complaint prediction features (DCNN). The prediction accuracy for heart disease is improved by this framework. The clinical dataset included data on BP, age, sex, difficulty breathing, cholesterol, blood sugar, and some other parameters for the prediction of cardiac disease. Classifying the sensor data that FNN has received allows for the identification of the heart’s status. The suggested method achieves an F1-score of 97%, an accuracy rate of 86.4%, and a precision of 76.2%. The performance assessment of this study, however, does not take into account crucial QoS metrics like power use, latency, etc. (Venkatesan et al., 2022).

In order to gather and analyze electrocardiogram (ECG) information from patients in order to quickly identify irregularities or heart attacks so that the proper therapies may be given, Minh et al. (2022) presented a fog-based IoT strategy in 2023. Using data mining techniques, this framework may extract pertinent information for the detection of cardiac illness at the network edge while maintaining the ECG data’s consistency by reducing noise. The framework not only ensures the data’s integrity but also improves the precision of real-time detection, as shown by practical studies. The processing of a patient request, however, is delayed significantly because to the time it takes for communication between nodes (Minh et al., 2022).

Verma et al. (2022) developed a Fog Enabled Technology for Clinical Healthcare System (FETCH), which incorporates edge computing devices with deep learning technology and automated monitoring to function on real-world healthcare systems, including those for heart disease and more. Fog Bus, which exhibits utility in regards to power use, internet bandwidth, jitter, latency, process completion time, and their correctness as well, is used by the recommended fog-enabled cloud computing architecture. Its computational efficiency is much increased, and the performance is optimised primarily for testing accuracy. High connection challenges that occur during data transfer are another key flaw with this architecture (Verma et al., 2022).

The system with smart healthcare for heart disease risk prediction described by Nancy et al. (2022) uses the fuzzy inference system and the bidirectional LSTM of the recurrent NN for the predictive task. Accuracy, precision, sensitivity, specificity, and F1-score of the suggested system are 98.85, 98.9, 98.8, 98.89, and 98.85%, respectively. For data collection, this intelligent system for predicting heart illness uses IoT devices, while the cloud is used for other crucial activities. Less time was needed for training using this framework, however it does not operate in a real-time setting (Nancy et al., 2022).

A reference layered communication framework was provided by Srinivasu et al. (2022) for the nodes and devices in real-time communication. The labor-intensive services are deliberated in this framework. The future of healthcare is presented by the feature-perspective elements of 6G technology, which allow for efficient treatment and sophisticated service integration. However, crowded network configurations also give rise to connectivity problems (Srinivasu et al., 2022).

Harris Hawk Optimizer (HHO), a recently developed robust optimizer, was the subject of Hussien et al. (2022) study. They concentrated on its applications and advancements. In regards to the new variations of the HHO algorithm and its many applications, the literature review study offers in-depth insight about potential future approaches and ideas worth investigating. However, it is not the purpose of this framework to create more real-time frameworks (Hussien et al., 2022).

Muttaqin et al. (2023) proposed the improved version of whale optimization algorithm by integrating the chaotic maps to tune the hyper parameters of Support vector machines (SVM) to achieve the better performance in the classification of cancer datasets. Basha et al. (2021) enhanced the exploitation and exploration phases of Harris hawk algorithm (HHA) by using chaotic maps to overcome the practical challenges in tuning the convolutional neural network (CNN) to classify the brain datasets. In Tair et al. (2022), the HHA ‘s exploration and exploitation phase is enhanced by using the chaotic maps to boost the convergence rate of HHA.

It is evident, though chaotic based HHA and other meta –heuristic algorithms are used for tuning the hyper parameters of several DL algorithms, still deployable and computational aware optimized models are badly needed for IoT-Edge-Fog Environment. Furthermore, existing works details about the chaotic maps in meta-heuristic algorithm but the selection of chaotic maps in combination with the meta-heuristic and DL models remains tricky and time- consuming process. Table 1 provides a quick summary of the literature review.

**Table 1:** Quick analysis of literature survey.

Authors	Techniques incorporated	Performance metrics analysis							Advantages	Disadvantages
		Accuracy	Specificity	Sensitivity	Precision	F1-score	Computational complexity	Time complexity		
Jenifer et al. (2022)	Edge base detection using IoT	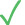 High	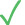 High	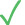 Average	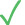 Average	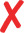 Low	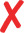 High	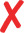 High	High learning rate	Not suitable for real time environment
Raju et al. (2022)	CCNN + GSO optimization	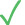 Average	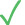 Average	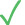 Average	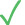 Average	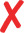 Low	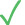 Average	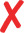 High	Supports real time environment	Prediction efficiency needs to be improvised
Gupta et al. (2023)	CNN	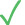 Average	Not analyzed	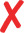 Low	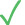 Average	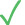 High	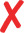 High	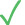 Average	Handle high scale datasets	More energy consumption
Sarmah (2020)	DLMNN	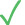 Average	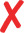 Low	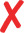 Low	Not analyzed	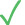 Average	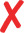 High	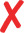 High	High security	Does not incorporated any optimization which increased the training time
Mansour et al. (2021)	CSO-CLSTM	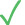 Average	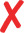 Low	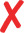 Low	Not analyzed	Not analyzed	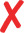 High	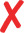 High	Required less energy consumption	Security of the IoT data were not considered
Lavanya et al. (2023)	DWT + CNN	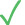 Average	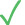 Average	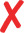 Low	Not analyzed	Not analyzed	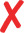 High	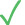 Average	Required less time for training	The system does not have compatibility with edge devices
Venkatesan et al. (2022)	FNN + DCNN	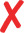 Low	Not analyzed	Not analyzed	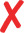 low	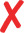 low	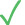 Average	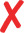 High	It required less attributes for training	The effectiveness of this technique in older people with persistent heart conditions has not been tested.
Minh et al. (2022)	Fog-based IoT approach	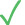 Average	Not analyzed	Not analyzed	Not analyzed	Not analyzed	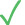 Average	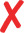 High	Guaranteed integrity of data and support real time environment	Required more attributes to achieve high accuracy
Verma et al. (2022)	FETCH	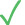 Average	Not analyzed	Not analyzed	Not analyzed	Not analyzed	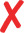 High	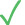 Average	Improves the computing efficiency	High Connectivity issues
Nancy et al. (2022)	Bi-LSTM	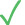 High	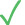 Average	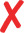 Low	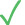 Average	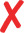 Low	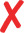 High	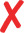 High	Required less timing for training	Not suitable for real time environment
Srinivasu et al. (2022)	6G Networks	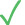 High	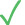 High	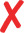 Low	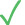 Average	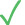 Average	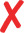 High	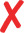 High	It required less attributes for training	High Connectivity issues
Hussien et al. (2022)	HHO	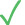 High	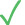 Average	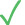 Average	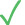 Average	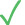 Average	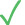 Average	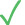 Average	Required less timing for training.	This framework does not covered more real time frameworks.
Muttaqin et al. (2023)	CNN	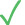 High	Not analyzed	Not analyzed	Not analyzed	Not analyzed	Not analyzed	Not analyzed	It required less attributes for training	Not suitable for real time environment
Tair et al. (2022)	Chaotic enabled Whale optimization	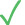 High	Not analyzed	Not analyzed	Not analyzed	Not analyzed	Not analyzed	Not analyzed	High accuracy	Needs improvisation for IoT environment
Basha et al. (2021)	Chaotic HHA	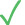 High	Acceptable	Acceptable	Not analyzed	Not analyzed	Not analyzed	Not analyzed	Improved performance over the other algorithms	Needs improvisation for IoT environment

## 3 Proposed methodology

**Figure 1:**
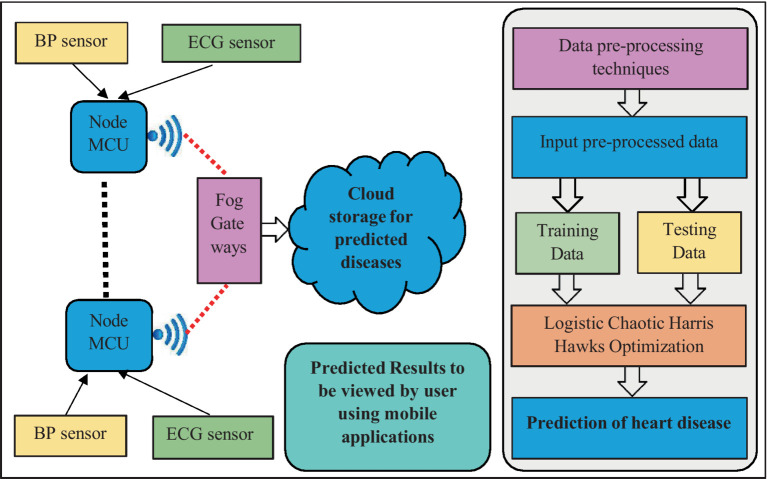
Complete structure of the suggested framework.

The Figure 1 provides the complete structure of the suggested framework which comprises of the three main software components: (i) Fog-IoT Data collection unit (FIDU) (ii) Data pre- processing (iii) Hybrid Deep Learning module.

### 3.1 Data collection unit

To evaluate how well the suggested model works, approximately 1,672 participants, ages 22 to 65, were chosen. About half of the participants are healthy, and the other 50% are averagely affected by heart disease. This research work uses IoT for collecting the heart signals from the volunteers. To collect the heart data, Electrocardiography (ECG) sensors are used and interfaced with NodeMCU and MCP3008. These boards are used to gather the subjects’ ECG signals and send them to adjacent edge devices running NVIDIA Jetson Nano Boards. When the 3.3 V batteries get low, they may be changed out with another set of batteries to power the IoT boards. Besides, the ECG data collection, blood pressure of the patient is also measured which will be used as the reference values to fulfill the missing values at the pre-processing stage. Moreover, WIFI enabled NVIDIA Jetson Nano Boards are used as Fog gateways and end output data will be stored in the AWS (Amazon Cloud Services) Cloud. Each IoT-BAN node which is mounted on the patients is identified as Identified Tags (ID). Each node transmits the ECG data and Blood pressure to the Fog gateways which is then used for the training the networks. Along with the ECG and BP, age and gender of subjects are also noted. Nearly 60 subjects with the age ranges from 22 to 65 were used for experimentation. Table 2 illustrates the sample format of data collected from the real time test beds. Table 3 presents the total number of data used for the testing and evaluation.

**Table 2:** Sample datasets collected from the real time IoT-BAN device networks.

Sl. no	Patients ID	Attributes			
		Age	Gender	ECG data	BP data
1	ID001	34	Male	223	130
2	ID002	41	Female	190	90
3	ID003	29	Male	67	110
4	ID004	59	Female	90	110
5	ID005	22	Female	91	89
6	ID006	24	Male	100	80
7	ID007	45	Female	129	90
8	ID008	30	Male	143	81
9	ID009	43	Male	189	120
10	ID010	46	Male	190	120

**Table 3:** Real time data used for the testing and evaluation.

Dataset description	No of data	No of records	No of attributes	Associated tasks
Real time datasets	1,672	300	04	Prediction

### 3.2 Data pre-processing and data preparation

In order to identify values for various aspects, a measure of the deep learning model’s input, the data received from common ECG sensors and blood pressure sensors must be pre- processed (Shou et al., 2021; Tair et al., 2022; Rajkumar et al., 2023). For this, the system needs to be supplied domain knowledge that is specific to the application. The data needed to be normalized because it was significantly skewed. The data for Blood Pressure (BP) is equally biased, and individuals with heart disease had higher BP readings (greater than 130/100) than those without heart disease. The distribution of the healthy patient’s heartbeats is leptokurtic, while the patient’s aberrant heartbeat levels also exhibit some target-specific behavior. Even at their highest heart rate, healthy persons have a much greater maximum heart (about 160) rate than people with heart disease (around 150). In this research article, Studentized Residual methods (Krishnan et al., 2021) are used to fill the missing values and non-meaningful data since these signals are subjected to the different environmental noises and EMI interference from the other devices. These pre- processed data are used for training and testing process that makes the decision of patient‘s heart condition.

### 3.3 Hybrid deep learning model

This module in the framework uses the pre-processed data for training the proposed hybrid learning model. As discussed, data are obtained from the BAN-IoT nodes mounted on the body of the patients which is then feed to the hybrid deep learning technique that consists of combination of self-attention based gated units with the Logistics Chaos based Harris Hawk optimised feed forward learning networks. The working principle of the complete learning framework is presented in the preceding section.

#### 3.3.1 Gated recurrent units

Most people find GRU to be the most fascinating form of long short-term memory. The idea presented by Waris and Koteeswaran (2022) suggests combining the forget gate and input vector into a single vector. This network supports long term sequences as well as long memories. The complexity is significantly lower when compared to that of the LSTM network.

The following Equations (1–4) show the characteristics of GRU.


(1) $$h_{t}=\left(1-x_{t}\right)⊙h_{t-1}+x_{t}⊙h_{t}$$

(2) $${\tilde{h}}_{t}=g\left(W_{h}x_{t}\right)+U_{h}\left(r_{t}⊙h_{t-1}\right)+b_{h}$$

(3) $$Z_{t}=\sigma \left(W_{h}x_{t}+U_{z}h_{t-1}+b_{z}\right)$$

(4) $$r_{t}=\sigma \left(W_{h}x_{t}+U_{z}h_{t-1}+b_{r}\;\right)$$

The general GRU characteristic equation is as follows:


(5) $$P=GRU\left(\overset{n}{\underset{t=1}{\sum }}\left[x_{t},h_{t},z_{t},r_{t}\left(W\left(t\right),B\left(t\right),\eta \left(\mathrm{tannh}\right)\right)\right]\right)$$

where xt⇨ input feature at the present state; yt⇨ output state; ht⇨ output of the module at the present instant; Zt and rt⇨ update and reset gates; *W*(t)⇨ weights; *B*(t)⇨ bias weights at present instant. As from preprocessed ECG data, these GRU networks are used for extracting the temporal properties. The GRU model uses the Chaotic Harris Hawk method to optimize the weights in the dense neural network of the GRU and may learn the R–R interval peak with larger modifications to prevent over-fitting problems. With the intention to improve feature extraction and address the complexity issue, this research effort introduces self-attention based gated units.

#### 3.3.2 Self-attention evoked gated recurrent units

The attention map was developed in 2014 to help define the concepts correctly in sequence- to-sequence architecture. Most recent research focuses on simulating redundant features that can aid in precise categorization procedures through the usage of attention layers. The intra- attention mechanism, also known as the self-attention mechanism, functions by producing the three vectors Q, K, and V for every input sequence. This results in the output sequences being created from the input sequences from each layer. Basically, it is a method that uses scaled dot functions to map the query to the set of key pairs. The following Equation (6) is used to get the dot product for self-attention mathematically:


(6) FKQ=KQT)/VK^0.5

GRU networks are developed by integrating the attention layers after each and every GRU cell in order to collect the relevant data from the BAN-IoT test beds. The GRU network collects many heart-related features that can be used to improve classification. However, these features include more non-redundant data (such noise signals) that could have an impact on training time, which adds to the overhead in the classification layer and delays decision-making. In order to get around this classification overhead, self-attention layers are inserted in between every single GRU cell. Equation (7) represents the attention features for single layered GRU and attention.


(7) $$Y=\mathrm{Softmax}\;\left(P\left(GRU\right),F\left(K,Q\right)\right)$$

Hence the overall structural representation of the Attention Evoked GRU cells is as follows with the Equation (8):


(8) $$Y\left(n\right)=\overset{L}{\underset{i=0}{\sum }}Y\left(i\right)$$

Where L = No of GRU cells.

### 3.4 Feed forward learning networks

The fully integrated feed forward network then receives these features for final categorization. The completely connected layers are constructed using the Extreme Learning Machines (ELM) idea. An ELM has a single hidden layer and is a type of neural network that operates on the auto-tuning property. ELM exhibits greater performance, fast speed, and low processing cost in comparison to all other learning methods like SVM, Bayesian Classifier, KNN and even RF.

This specific neural network has just one hidden layer, which rarely requires to be adjusted. Compared with other machine learning algorithms like SVM and RF, ELM performs better, faster, and with less overhead. ELM yields better accuracy as well as improved performance using the kernel function. Higher approximation and reduced training error are the key advantages of the ELM. Considering that ELM makes use of automatic weight bias adjustment and non-zero activation functions. The ELM’s intricate operating mechanism is covered in Alwateer et al. (2021). After Attention maps, the representation of the inputs features mapping to the ELM is represented used the Equation (9)


(9) $$X\left(m\right)=\overset{L}{\underset{k=0}{\sum }}Y\left(k\right)$$

Where Y⇨ features from Self Attention GRU network, The yield ELM function’s symbol is.


(10) $$Y\left(m\right)=X\left(m\right)\beta =X\left(m\right)X^{T}{\left(\frac{1}{C}XX^{T}\right)}^{-1}0$$

### 3.5 Hyper parameter tuning of the feed forward learning networks

Tuning hyper-parameters is done to obtain the best hyper-parameters for the suggested model in order to further reduce complexity. Prior to model training, hyperparameter adjustment is done. Hyperparameters that need to be modified in this study are the number of hidden layers, number of hidden units, dropout rate, epochs, and batch size. In this research, Logistic Chaotic Harris hawk Optimization algorithms are used for the tuning the parameters of the networks for the better classification. The principle of operation of logistic chaotic Harris hawk optimization algorithms is presented below.

#### 3.5.1 Harris hawk optimization algorithm

**Figure 2:**
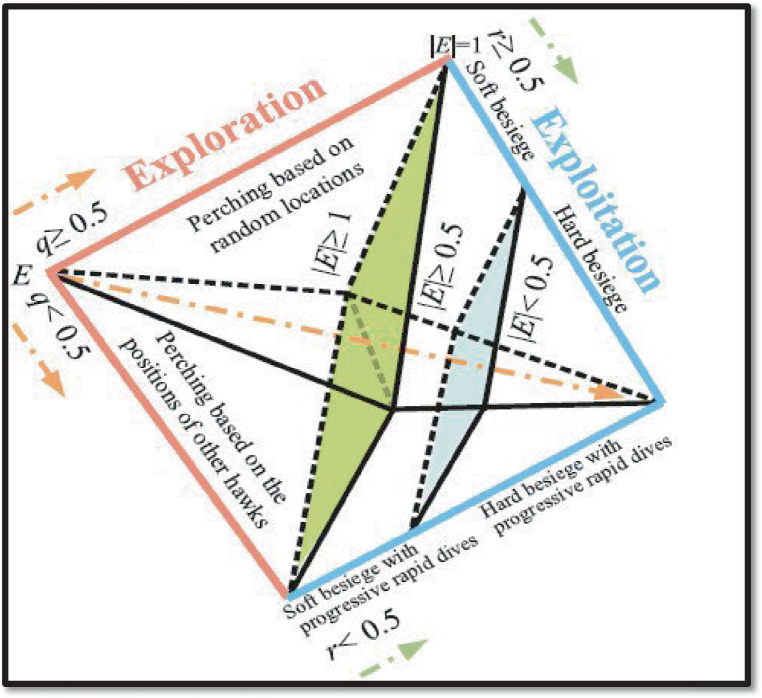
Different HHO phases.

The various ways that hawks explore and attack their prey provide as inspiration for the HHO algorithm (Waris and Koteeswaran, 2022). Three stages make up HHO, a population-based optimisation technology: exploration, transformation of exploration, and exploitation. The different phases of HHO are shown in Figure 2.

##### 3.5.1.1 Exploration stage

The relative positions of rabbits or other members are used to determine the hawks’ perching spots at this stage, which are modelled as follows:


(11) $$\begin{array}{l} X\left(t+1\right)=\\ \left\{\begin{array}{c} X_{\mathrm{rand}}\left(t\right)-r_{1}|X_{\mathrm{rand}}\left(t\right)-2r_{2}X\left(t\right)|,\;q\geq 0.5,\\ \left(X_{\mathrm{rabbit}}\left(t\right)-X_{m}\left(t\right)\right)-r_{3}\left(LB+r_{4}\left(UB-LB\right)\right),\;q<0.5\text{,} \end{array}\right\} \end{array}$$

(12) $$X_{m}\left(t\right)=\frac{1}{N}\overset{N}{\underset{i=1}{\sum }}X_{i}\left(t\right)\text{,}$$

Where *X*_rabbit_ (t) is the prey’s position, *X*(t) is the hawks’ position, and *X*(t + 1) is the new position of the hawks in the Equation (11). The absolute value of the elements is denoted by the modulus. In the interval (0, 1), the random numbers 𝑟_1_,_2_, 𝑟_3_, 𝑟_4_ and 𝑞 are found. UB and LB are the upper and lower bounds of variables. 𝑋_𝑟𝑎𝑛𝑑_ (𝑡) is the position of a random hawk population. The average position of the hawk population at any given time is 𝑋_𝑚_ (𝑡) calculated using the Equation (12).

##### 3.5.1.2 Transformation of exploration and exploitation

The following Equations (13 14) are used to evaluate the prey’s escape energy, which plays a significant role in the transition stage:


(13) $$E_{1}=2\left(1-\frac{t}{T}\right)$$

(14) E=E0E1

Where *T* is the maximum number of iterations; E0 represents the initial energy of a prey, randomly varying between −1 and 1; and *t* is the current iteration.

##### 3.5.1.3 Exploitation stage

At this stage, the prey is attacked by the hawks use all four of their methods as well as its escape mechanism. A successful capture requires both escaping energy (*E*) and escape potential (*r*).

In the following equations, Hawks executed a light besiege when *r* ≥ 0.5 and |E| ≥ 0.5. This indicates that the prey has sufficient energy but has failed in its attempt to escape.


(15) $$X\left(t+1\right)=\Delta X\left(t\right)-E|JX_{\mathrm{rabbit}}\;\left(t\right)-X\left(t\right)|$$

(16) $$\Delta X\left(t\right)=X_{\mathrm{rabbit}}\;\left(t\right)-X\left(t\right)$$

Where 𝑋_𝑟𝑎𝑏𝑏𝑖𝑡_ (𝑡) denotes the leap strength, which varies randomly with each in Equations (15 16), and

∆(𝑡) indicates the difference between the prey’s current position and the hawks’ position at iteration *t*. The random number 𝑟_5_ ranges from 0 to 1.

Hawks attempt to escape by applying a hard besiege on prey that has low escaping energy, as shown by *r* ≥ 0.5 and |*E*| < 0.5, which can be modelled as follows in the Equation (17):


(17) $$X\left(t+1\right)=X_{\mathrm{rabbit}}\;\left(t\right)-E|\Delta X\left(t\right)|$$

When *r* < 0.5 and |*E*| ≥ 0.5, hawks execute progressive fast dives, modelled as follows in Equation (18), to hunt through a more cunning soft encirclement termed soft besiege.


(18) $$Y=X_{\mathrm{rabbit}}\;\left(t\right)0v-E\;|X_{\mathrm{rabbit}}\;\left(t\right)-X\left(t\right)|$$

(19) $$Z=Y+\mathrm{SXLF}\;\left(D\right)$$

Where 𝐷 be the dimension of the problem, 𝑆 be a random vector of size 1 × 𝐷, and LF be the Levy flight function as specified by the given Equations (19-21).


(20) LFd=0.01×u×σv1β′

(21) σ=Γ1+β×sinπβ2Γ1+β2×β×2β−121β

Where *Γ* is a typical Gamma function, *β* is a constant that has a bound of 1.5, and *u*, *v* are random normal distribution vectors of size 1 × *d*. The positions of the hawk can be modelled by using the Equation (22)


(22) $$X\left(t+1\right)=\begin{array}{c} Y\;\mathrm{if}\;F\left(z\right)<F\left(X\left(t\right)\right)\\ Z\;\mathrm{if}\;F\left(z\right)<F\left(X\left(t\right)\right) \end{array}$$

A hard besiege is declared when the prey’s energy is exhausted (*r* < 0.5 and |*E*| < 0.5). Equations (23) and (24), representing *Y* and *Z*, are used in the calculation.


(23) $$Y=X_{\mathrm{rabbit}}\;\left(t\right)-E|JX_{\mathrm{rabbit}}\;\left(t\right)-X_{m}\left(t\right)|$$

(24) $$Z=Y+S\times LF\left(D\right)$$

The updating method is as follows:


(25) $$X\left(t+1\right)=\begin{array}{c} Y\;\mathrm{if}\;F\left(z\right)<F\left(X\left(t\right)\right)\\ Z\;\mathrm{if}\;F\left(z\right)<F\left(X\left(t\right)\right) \end{array}$$

### 3.6 Logistic chaotic Harris hawk optimization algorithm (LC-HHOA)

**Figure 3:**
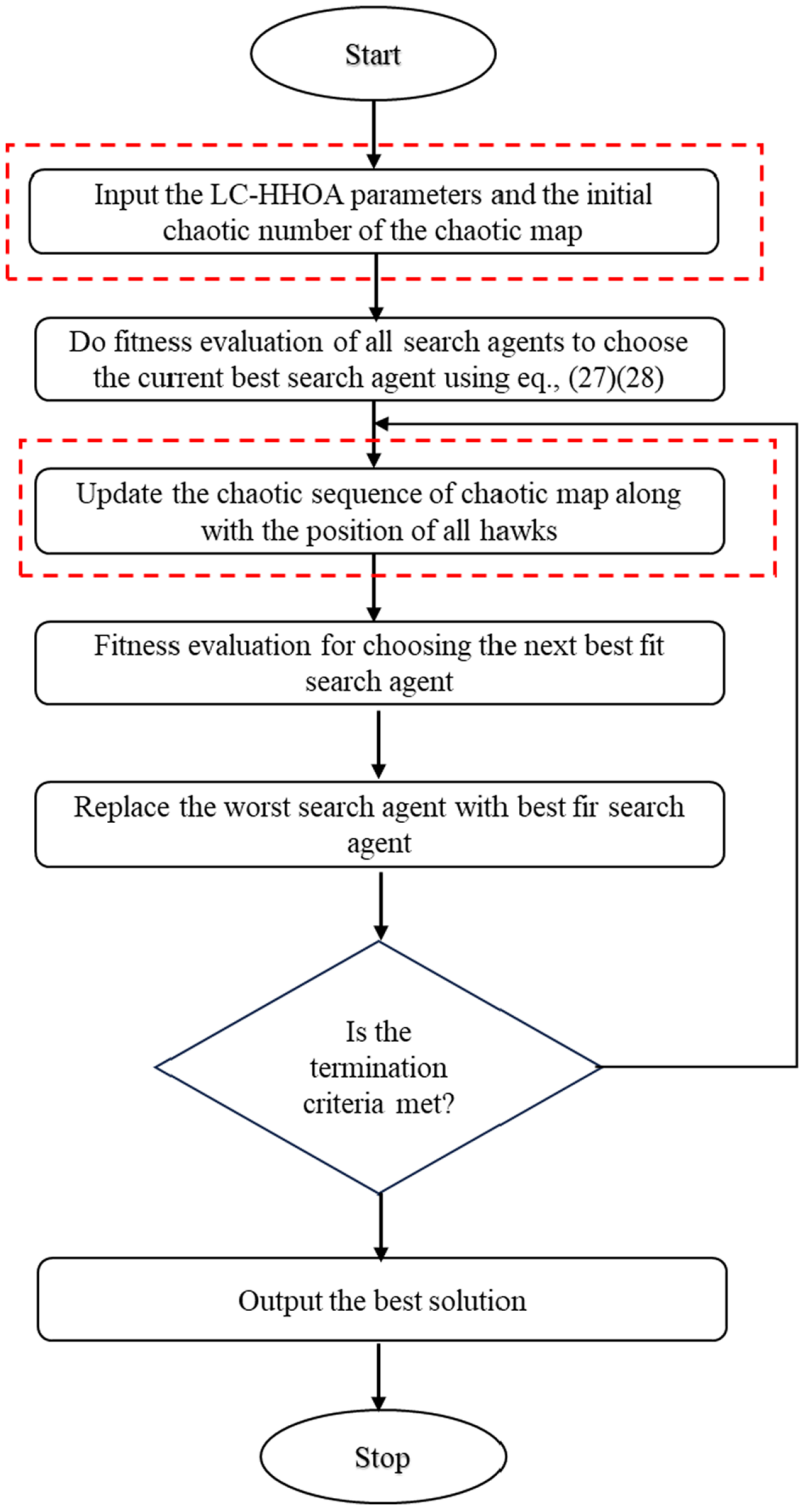
Flow chart for the proposed model LC-HHOA.

Even though Harris hawk has a higher rate of convergence than other algorithms, it still requires improvisation to find the overall optimal solution, which lowers the rate of convergence. So to increase the efficiency of the Harris hawk algorithm, Logistic Chaotic maps are introduced. The detailed descriptions of Logistic Chaotic Maps are discussed in Alwateer et al. (2021). The logistic maps are mainly selected due to its simple and easily deployable. Due to the dynamic behavior and unpredictable nature of these maps, exploration search space is increased in which the trapping problem is prevented in the local minima. Probability distributions are used to create unpredictability in practically all meta-heuristic techniques with stochastic elements. Figure 3 presents the complete flow diagram for the proposed optimization algorithm.

The incorporation of Logistic Chaotic maps in Harris hawk optimization mathematically expressed by converting the random search variables into logistic maps in rabbit searching mechanism. The chaotic variables *X* chaos are used in place of ∆*X* and *X*(t) in rabbit searching mechanism. The mathematical expressions for representing the chaos based positions in the Harris hawk searching mechanism is given as follows in the Equations (26 27).


(26) $$X\left(t+1\right)=X_{\mathrm{chaos}}-E|JX_{\mathrm{rabbit}}\left(t\right)-X_{\mathrm{chaos}}|$$

(27) $$X_{\mathrm{chaos}}=X_{\mathrm{rabbit}}\;\left(t\right)-X_{\mathrm{chaos}}$$

Where *X*_rabbit_ (t) is the leap strength that occurs randomly at each iteration and *X*_chaos_ is the difference between the prey’s current position and the hawks’ position at chaotic iteration *t*. The random number r_5_ ranges from 0 to 1.

### 3.7 LC-HHOA based feed forward training networks

The weights of the dense networks employed by GRU are optimized using the simple LC- HHOA, as was mentioned in Section 3.3.1. The GRU training system retrieves the randomly selected hyperparameters at first. The fitness value of the recommended method is given in Equation (28). Hyperparameters are computed using Algorithm 1 for each iteration. Whenever the fitness function agrees with the Equation (28), iteration ends.


(28) $$\begin{array}{l} \mathrm{Fitness}\;\mathrm{Function}=\\ \left\{\begin{array}{l} \max\left(\mathrm{Accuracy}\right)+\max\;\left(\mathrm{Precision}\right)+\\ \max\left(\mathrm{Recall}\right)+\max\left(f1-\mathrm{score}\right) \end{array}\right\}/\mathrm{No}\;\mathrm{of iteration} \end{array}$$

The developed classification layer quickly and with minimal computation detects normal and heart disease conditions after the input Algorithm 1 presents the suggested classification layers’ operational mechanism. Hence the Self-attention evoked GRU units are used to extract the R–R intervals followed by the LC-HHOA dense training networks for an effective prediction of the heart diseases. Based on the proposed LC-HHOA, 10 GRU cells, 221 hidden nodes, momentum of 0.01, epochs of 140 are selected.

#### ALGORITHM 1

**Table:** 

Steps	Algorithm-1 // Pseudo Code for the Proposed Model
01	Input: Bias weight, concealed units, Epochs, Learning Rate
02	Target: Prediction of Normal/Heart Diseases
03	Bias weight, concealed units, epochs, and learning rate should be assigned at random.
04	Set the three parameters
05	Use While loop for true
06	Use equation (5) to determine the output from GRU cells
07	Use the formula (28) for determining the fitness function
08	Start the For loop from t=1 to Max. iteration
09	Use equations (25 & 26) to assign the bias weights & input layers
10	Use equation (28) for calculating the fitness function
11	Check If condition for (Fitness function is equal to threshold)
12	jump to Step 17
13	Otherwise
14	jump to Step 8
15	Stop
16	Stop
17	Calculate the Output function from Dense layers using Equation (10)
18	If Output function is equal to 1
19	//Normal or No disease is determined ⇨estimated
20	Else if (output function<=2&&>1)
21	// heart disease -1 ⇨estimated
22	Else if (output function <=3&&>2)
23	// heart disease -2 ⇨estimated
24	Else
25	jump to Step 9
26	Stop
27	Stop
28	Stop

## 4 Implementation

### 4.1 Hardware setup

**Figure 4:**
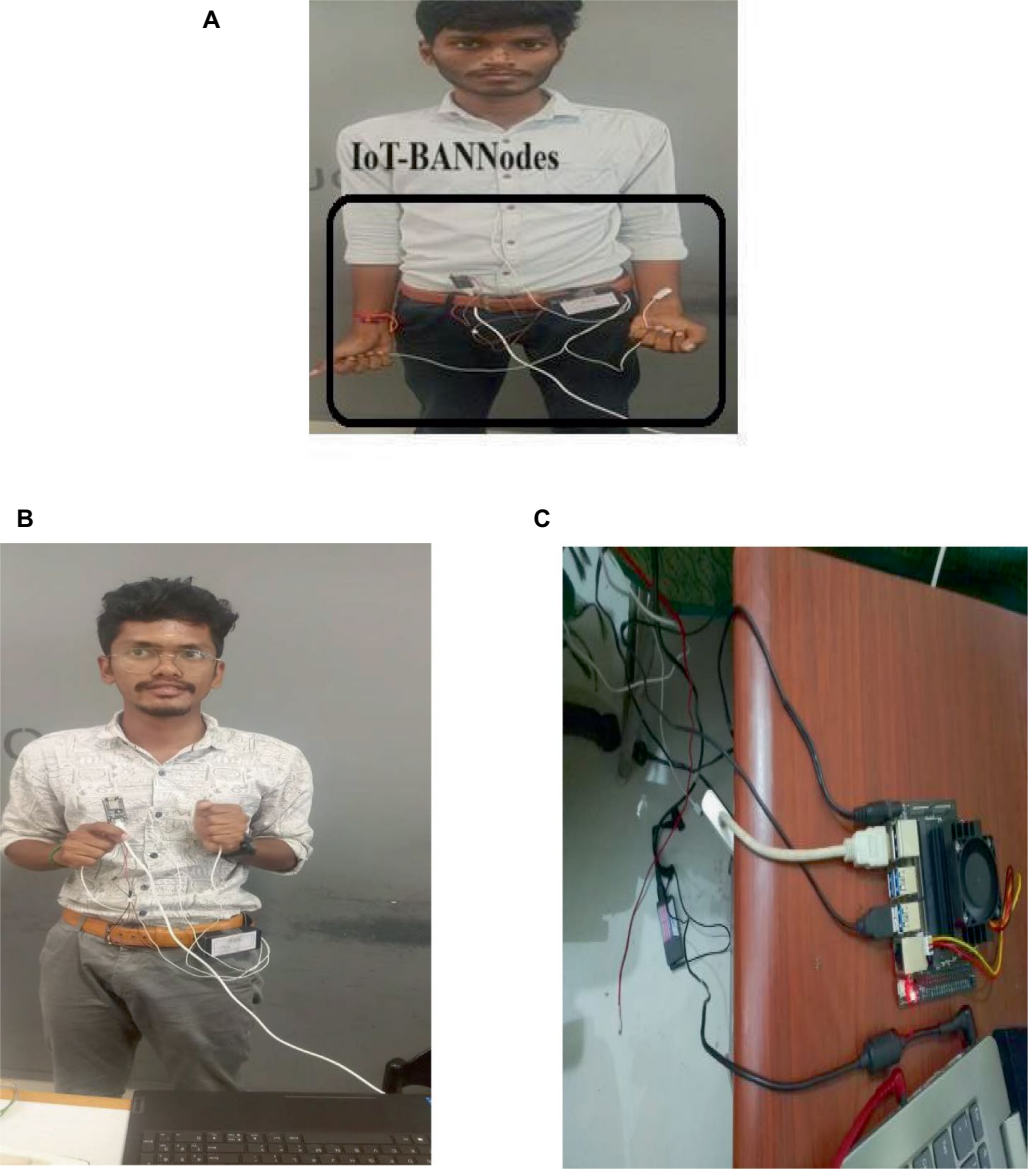
Experimental setup **(A,B)** IoT-BAN nodes interfaced with ECG and BP Sensors Mounted on the Subjects. **(C)** Fog gateway used with the NVIDIA Jetson Nano Boards.

The proposed model was implemented using Python-based Keras Libraries, Tensorflow v2.1 as the backend, and NVIDIA Jetson Nano V4. MCP3008 interfaced NodeMCU is used as BAN-IoT node for collecting the ECG and Blood pressure data from the different Subjects. Nearly 5 IoT nodes with Fog gateway is used for the experimentation. Table 4 presents the hardware specification of the BAN-IoT nodes implemented for the ECG and BP measurements. Figures 4A–C details about the experimentation of proposed model using IoT- BAN nodes and Fog Environment. Figure 4C shows the NVIDIA based Fog Gateway used in experimentation. AD3282 ECG sensor is used for measuring the ECG signals whereas Omron 2SMPP-02 Blood pressure sensor is deployed for measuring the BP.

**Table 4:** Hardware specification for the BAN-IoT nodes used for the experimentation.

Sl.no	Hardware descriptions	Specifications
1	Microcontroller in BAN-IoT nodes	NodeMCU
2	Gateways	NVIDIA Jetson Nano
3	Analog –to –digital channels resolution	10-bits
4	ECG sensor	AD3282
5	BP sensor	2SMPP-02
6	Power	3.3 V
7	Mode of connectivity	WIFI enabled devices

### 4.2 Dataset details

To train and test the suggested model, about 1,672 datasets were gathered and provided. Eight hundred and eighty-seven were healthy and remaining were unhealthy. The complete dataset were split into different testing and training ratios to achieve better performance of the proposed model.

## 5 Performance evaluation

Performance measures like accuracy, precision, recall, specificity as well as F1-score are evaluated (Ramasamy et al., 2021; Sarosh et al., 2022) and immediately compared with other cutting-edge deep learning algorithms employing a fog computing field to show the superiority of the proposed model. All the performance measures as well as latency overhead are also evaluated to demonstrate that the suggested model seems to be more productive and have low overheads. Table 5 presents the mathematical formula utilized to determine the performance measures. The early stopping technique is used to get around the generalization and overfitting issues. When the suggested model’s validation performance does not become better over time, this technique is utilized to end the iteration.

**Table 5:** Performance measures employed in the evaluation.

SL.NO	Performance measures	Expression
1	Accuracy	$\frac{TP+TN}{TP+TN+FP+FN}$
2	Recall	$\frac{TP}{T\;P+FN}\times 100$
3	Specificity	$\frac{TN}{TN+FP}$
4	Precision	$\frac{TN}{TP+FP}$
5	F1-Score	$2\frac{\mathrm{Precison}*\mathrm{Recall}}{\mathrm{Precison}+\mathrm{Recall}}$

TP and TN are true positive and negative, FP and FN are false positive and negative.

Predication case is defined in four parts: the first one is true positive (TP) in which the values are identified as true and, in reality, it was true also. The second one is false positive (FP) in which the values identified are false but are identified as true. The third one is false negative (FN) in which the value was true but was identified as negative. The fourth one is true negative (TN) in which the value was negative and was truly identified as negative.

### 5.1 Ablation study

**Figure 5:**
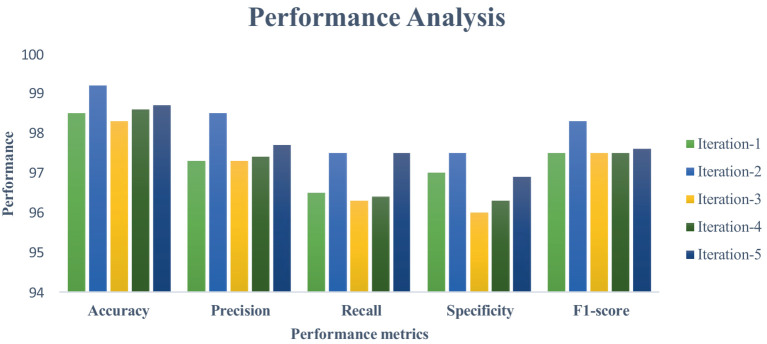
Validation performance of the proposed model employing Standard 70:30 training and testing datasets.

**Figure 6:**
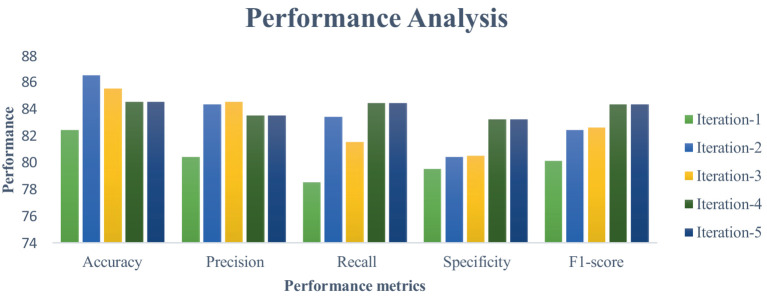
Validation performance of the RNN using Standard 70:30 training and testing datasets.

**Figure 7:**
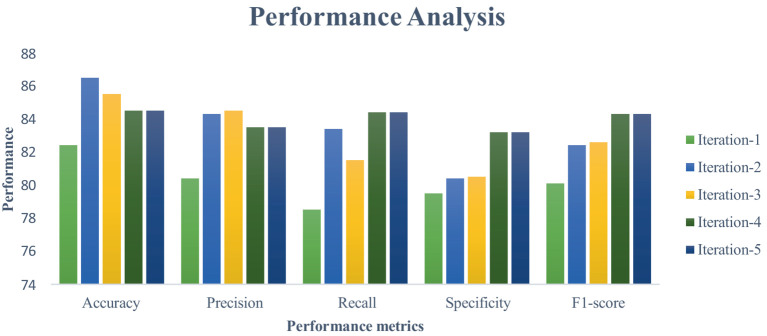
Validation performance of the LSTM using Standard 70:30 training and testing datasets.

**Figure 8:**
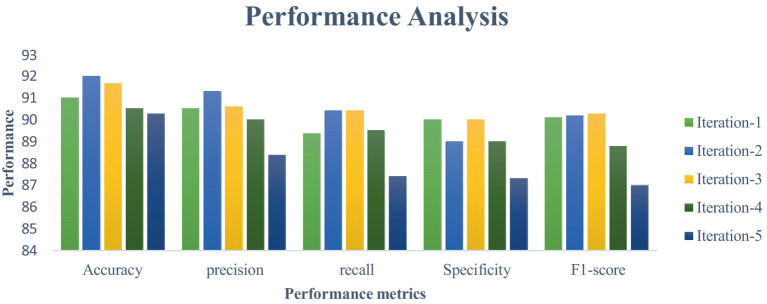
Validation performance of the GRU using Standard 70:30 training and testing datasets.

This section measures and compares many metrics to the most recent deep learning algorithms, including predictive accuracy, model building time, latency characteristics, and energy consumption. The deep learning techniques are considered for the experimentation are Recurrent Neural Networks (Devi et al., 2013), Long Short Term Memory (Priyanga et al., 2021), and Gated Recurrent Units (Liu and Kim, 2018). As the first step, 5-cross validation is conducted for each algorithm and performance is analyzed. In the scenario, the average performance of the suggested framework has performed better than the competition in Fog environment. The evaluation effectiveness of the various algorithms is displayed in Figures 5–8 using five –fold validation matrix. It is evident from Figures 5–8 that perhaps the proposed system has demonstrated superior efficacy in predicting cardiac illnesses.

**Figure 9:**
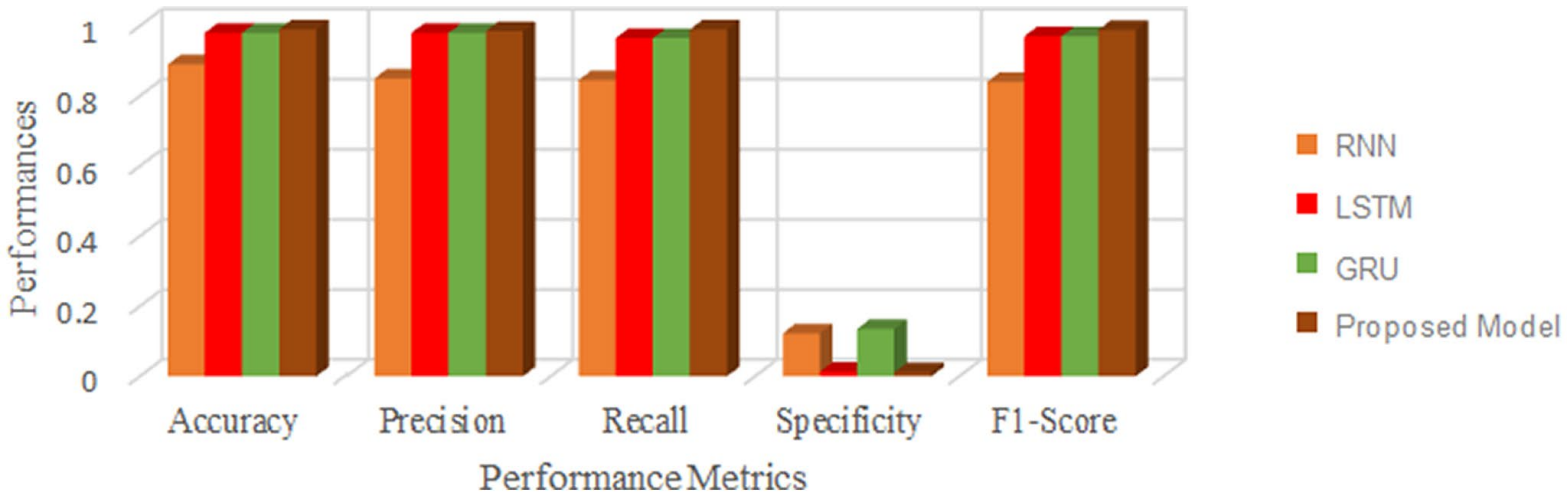
Comparison of the suggested deep learning algorithm’s performance with the test datasets = 20%.

**Figure 10:**
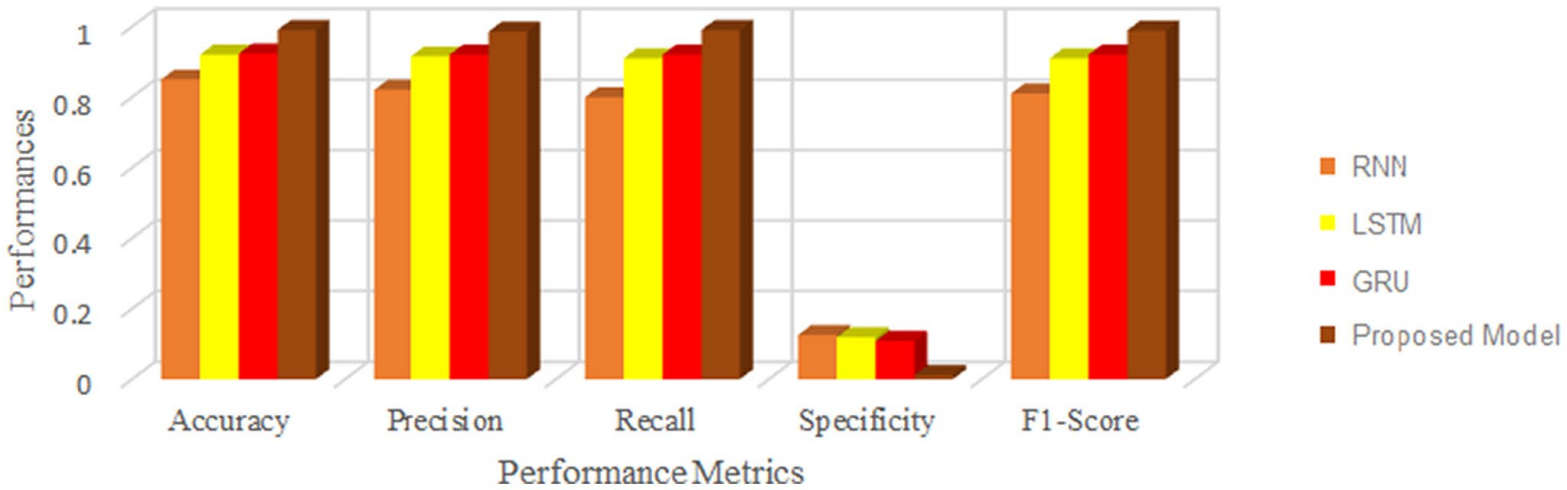
Comparison of the suggested deep learning algorithm’s performance with the test datasets = 30%.

**Figure 11:**
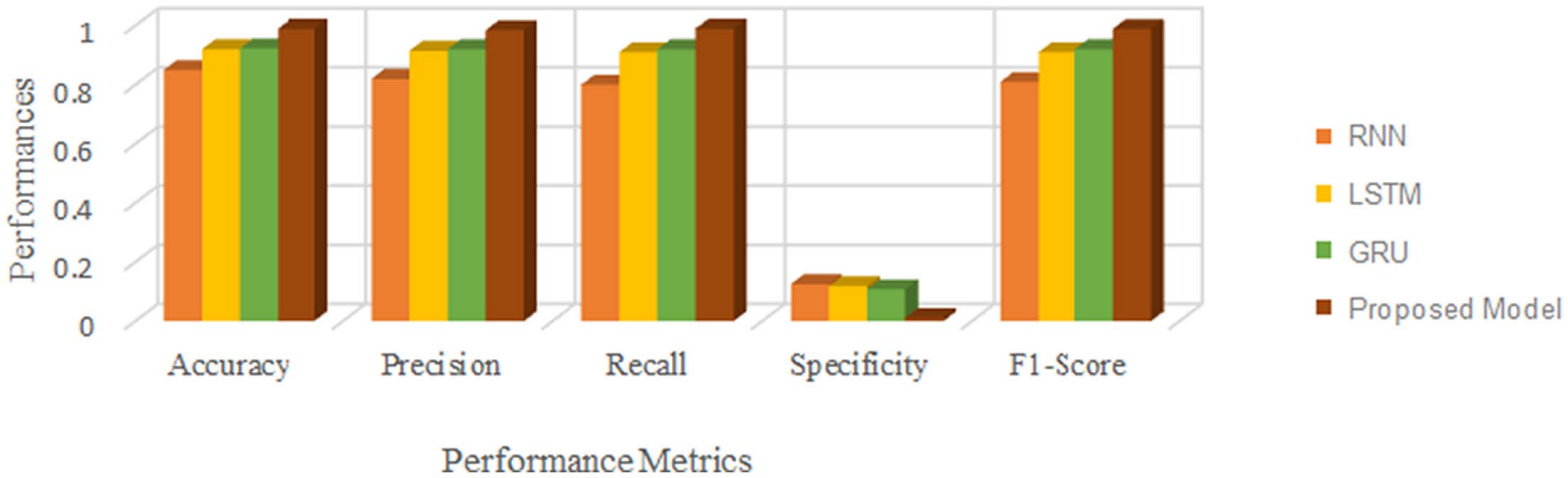
Comparison of the suggested deep learning algorithm’s performance with the test datasets = 40%.

Figures 5–8 provides the validation representation of deep learning model in Fog environment via usage of real-time datasets. For every iterations, average performance of the proposed model ranges from 99.5 to 99.7% whereas the other algorithms has produced little degraded performances ranges from 81% to 85. From these experiments, it is clear that the suggested model performs exceptionally well when it comes to predicting cardiac disease in the Fog-BAN environment. Figures 9–11 illustrates the comparative evaluation of the effectiveness of the various deep learning architecture employed in Fog-gateways with the different iterations of datasets.

Figure 9 illustrates how the RNN and LSTM models produced the weakest prediction performances, the suggested model and GRU models produced the most promising results in terms of heart disease prediction. From Figures 9–11, it is evident that the performance of the other existing algorithm degrades slowly as the number of testing datasets increases while the suggested model has maintained the steady efficiency even though datasets increases. Also, it is clear that when the number of datasets rises from 20 to 40% respectively, the suggested framework and GRU have generated performance that is remarkably similar. But the proposed algorithm has outperformed that the GRU when the number of datasets reaches to 40% which is evident from Figure 11. Overall, these results show that the most promising performance in predicting heart disease in a fog environment has been achieved by the suggested model, which combines feed forward networks optimized by Chaotic Harris Hawk with self-attention-based gated recurrent networks. To prove the superiority of the proposed model, several meta heuristic algorithms such as particle swarm optimization (PSO), ant colony optimization (ACO), Bat optimization (BAT), FireFly Optimization (FFO), reptile search algorithm (RSA), Spotted Hyena Optimization (SHO), and Harris Hawk Optimization (HHO) are combined with the GRU networks and its performance are evaluated and analyzed. Table 6 presents the classification performance of different models.

**Table 6:** Classification performance of the different combination of meta-heuristic models in GRU.

Algorithm	Accuracy	Precision	Recall	Specificity	F1-score
GRU + PSO	0.7289	0.712	0.702	0.703	0.702
GRU + ACO	0.72	0.702	0.69	0.70	0.70
GRU + BAT	0.75	0.734	0.72	0.71	0.72
GRU + FF	0.78	0.723	0.71	0.72	0.70
GRU + RSA	0.8499	0.82	0.812	0.80	0.82
GRU + SHO	0.86	0.812	0.802	0.81	0.802
GRU + HHO	0.89	0.843	0.820	0.82	0.834
GRU + LCHHOA (proposed model)	0.986	0.973	0.956	0.965	0.970

From the Table 6, it is clear that proposed combination has performed better than the other models due to the logistic integration of the different models.

### 5.2 Model building time analysis

Model Building Times (MBT) for various models across gathered datasets are shown in Table 7 with respect to validation. The main reason for evaluating MBT is because training time is an important factor to consider before a model can accurately diagnose cardiac diseases. This will have an immediate influence on the resources used and the performance of the model. Hence, MBT aids in establishing a good balance amongst computational burden and classifier performance. The average MBT for training the suggested model is 1.16 s, as shown in the table below, while GRU takes 1.56 s, LSTM takes 2.08 s, and RNN takes 2.45 s. It is clear from the analysis that the suggested model consumes only 1.16 s and finds its strong place in designing an effective monitoring system for the cardiac diseases.

**Table 7:** Model building time analysis for the different algorithms using real time datasets.

Training:	(MBT)-secs			
Testing dataset	RNN	LSTM	GRU	Proposed model
50:50	1.73	1.62	0.90	0.63
60:40	2.00	1.89	1.34	0.89
70:30	2.90	2.25	1.92	1.34
80:20	3.20	2.56	2.09	1.78
Average MBT	2.45	2.08	1.56	1.16

### 5.3 Experimental outcome analysis

Following the performance evaluation, the outcomes of the various models has been statistically validated. The comprehensive analysis of different models is determined and compared with their own advantages and disadvantages. Addition to the other existing deep learning models, classification outcomes based on the fitness function evaluations over 50 independent runs are illustrated in terms of best, worst, mean, median, standard deviations (SD) and variance are analyzed to prove the stability of the models. Further, indicator function outcomes over 50 runs with the relative stability parameters are experimented. The classification outcomes of the different models with the above mentioned parameters with its stability indicators are shown in Tables 8, 9, respectively.

**Table 8:** Fitness function based outcomes for the different combinations of GRU.

Algorithm	Best	Worst	Mean	Median	SD	Variance
GRU + PSO	0.73490	0.65483	0.72010	0.023912	0.067272	5.3 × 10^−6^
GRU + ACO	0.71230	0.63490	0.69034	0.019024	0.075343	6.3 × 10^−6^
GRU + BAT	0.75630	0.62390	0.65372	0.034930	0.06536	4.23 × 10^−5^
GRU + FF	0.78945	0.67890	0.71239	0.029043	0.0534102	3.130 × 10^−4^
GRU + RSA	0.85290	0.71012	0.73402	0.033393	0.053242	2.902 × 10^−4^
GRU + SHO	0.86342	0.70234	0.74022	0.045302	0.048933	2.7829 × 10^−4^
GRU + HHO	0.89267	0.80239	0.79340	0.059030	0.045672	2.0839 × 10^−4^
GRU + LCHHOA (proposed model)	0.98673	0.81202	0.85640	0.068313	0.0398303	1.28930 × 10^−4^

**Table 9:** Indicator function based outcomes for the different combinations of GRU.

Algorithm	Best	Worst	Mean	Median	SD	Variance
GRU + PSO	0.068932	0.005430	0.006690	0.00673	0.0002310	6.48 × 10^−7^
GRU + ACO	0.06734	0.005372	0.006993	0.006890	0.0002402	5.34 × 10^−6^
GRU + BAT	0.064320	0.0052930	0.007093	0.006903	0.0002406	5.893 × 10^−5^
GRU + FF	0.068230	0.0049033	0.0070234	0.007045	0.0002403	6.003 × 10^−5^
GRU + RSA	0.067830	0.0048934	0.007008	0.0070213	0.000289	6.1290 × 10^−5^
GRU + SHO	0.068003	0.0045363	0.007102	0.0070231	0.000310	5.894 × 10^−4^
GRU + HHO	0.068647	0.004890	0.007239	0.0071902	0.0003002	5.9034 × 10^−4^
GRU + LCHHOA (proposed model)	0.069802	0.0049083	0.00739	0.0072310	0.0003201	6.904 × 10^−4^

**Figure 12:**
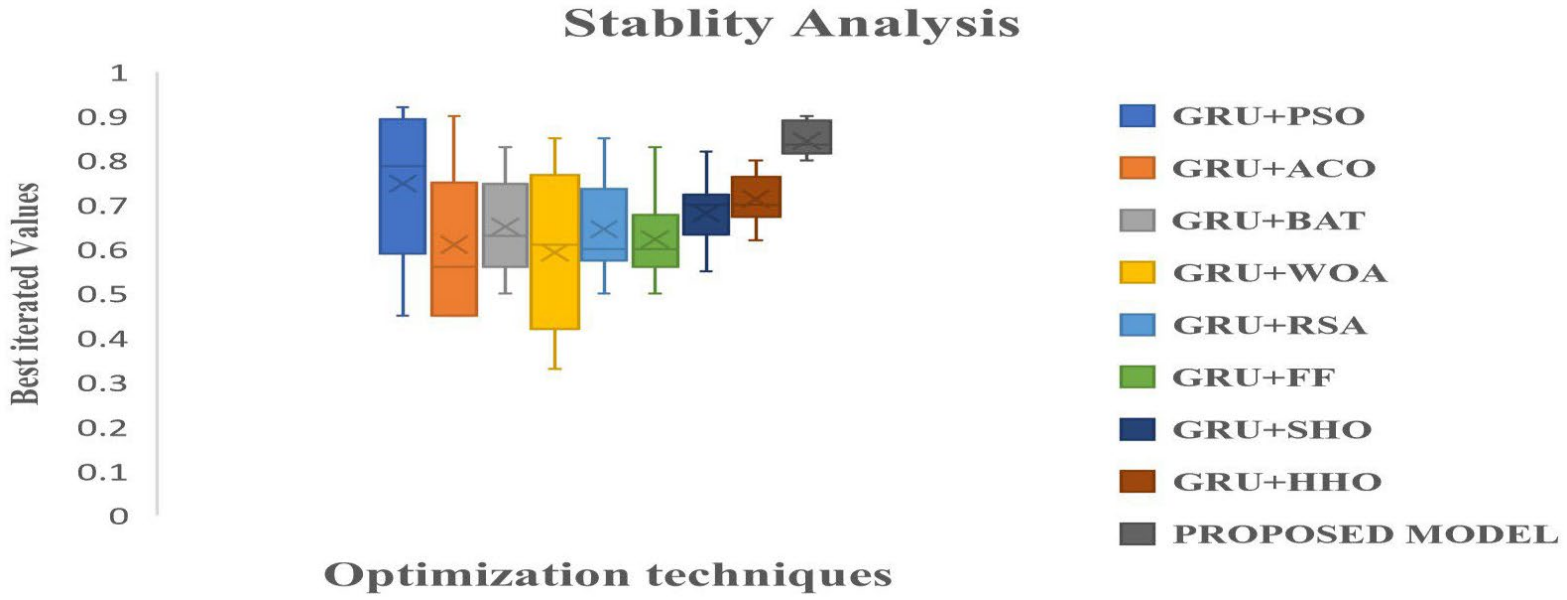
Box-analysis of the different optimization techniques integrated with the GRU networks.

Tables 8, 9 presented the outcome results from the different combinations of GRU networks. From the above tables, it is evident that the proposed model has produced the best outcome in comparison with the other optimization algorithm. The algorithm stability can be observed in the Figure 12. From the Figure 12, it is clear that the proposed model has produced the best outcome when compared with the other existing models.

**Figure 13:**
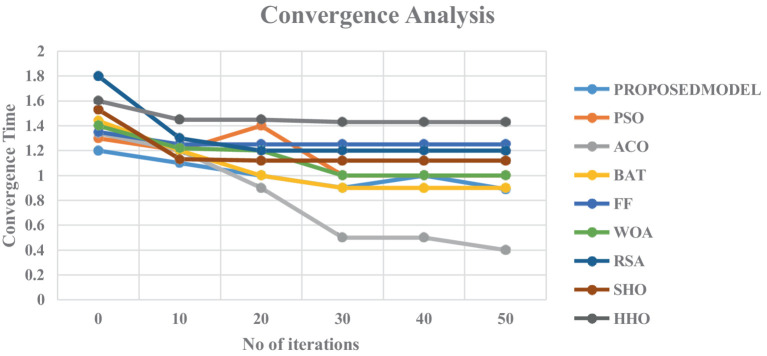
Convergence analysis for the different optimization algorithms.

The convergence study of the proposed scheme in compared with the above mentioned schemes for ranging iterations is presented in Figure 13. From the Figure 13, it is evident that proposed optimization is grown slightly over the other optimization algorithms. Though the HHO has demonstrated the best performance over the other traditional algorithms, but integration of chaos on HHO has produced the superior performance and finds its best place for tuning the hyper-parameters of the dense training networks.

To repeat the stability of the proposed model, hyper parameters chosen by the different optimization models are presented in Table 10.

**Table 10:** Hyper parameter selection by the different optimization algorithms.

Algorithm	No of epochs	Hidden layers	Learning rate	Batch size	Max depth
GRU + PSO	392	110	0.002	60	9
GRU + ACO	321	112	0.002	59	9
GRU + BAT	290	109	0.002	58	12
GRU + FF	234	106	0.002	57	10
GRU + RSA	230	103	0.0010	57	9
GRU + SHO	229	102	0.0001	56	8
GRU + HHO	222	090	0.0001	54	8
GRU + LCHHOA (proposed model)	200	084	0.0001	45	6

### 5.4 Statistical outcome validation process

**Figure 14:**
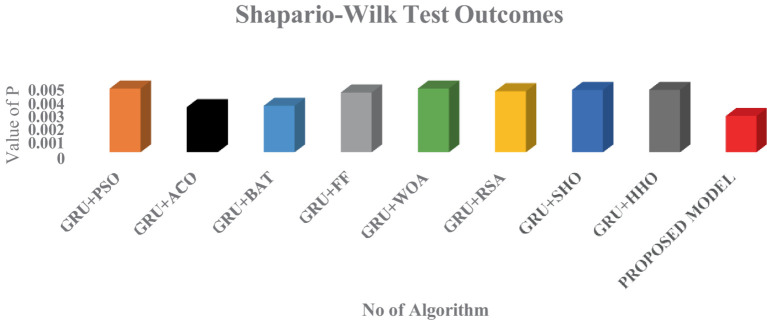
Statistical analysis of the different optimization algorithms in GRU.

Recently, meta heuristic algorithm in DL optimization demands the outcomes to be statistically validated. To establish the statistically supremacy of the models, various statistical tests are carried out for the different models. For an effective validation of the different models, three statistical criteria has to be met: independence, normality and homoscedasticity. The normality criteria is assessed using the Shapiro–Walk Outcomes as shown in Figure 14.

**Figure 15:**
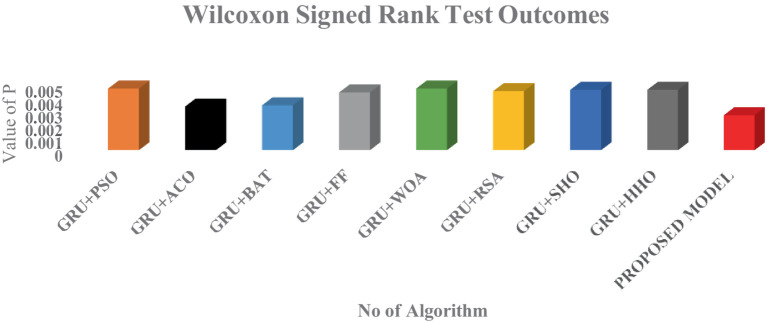
Wilcoxon signed rank algorithms for the different protocols.

As shown in Figure 14, all the models have produced the *p* which is less than 0.05 in which the normality test has been passed by all the models. But the proposed model has outperformed all the other existing models and proves its statically superior than the other optimization technique. Furthermore, to add the fuel in the performance of the model, Wilcoxon Signed – Rank test has been deployed. This test can be applied to the same data series consisting of the best outcomes achieved in every run of each metaheuristics. The outcome of each and every technique can be visualized in Figure 15.

From the above two figures, its is every clear that logistic chaos based Harris hawk optimization algorithm has outperformed the other models with the lowest *p-*value.

## 6 Conclusion and future scope

In the context of this study, a novel intelligent fog-based healthcare system is developed, which applies fog computing to heart patients and employs deep learning models to diagnose cardiac issues. Using coupling Chaotic Harris Hawk Optimized Feed Forward layers and Self Attention Gated Recurrent Units, the proposed framework combines real-time data collecting with robust deep learning architectures. Accuracy, precision, recall, specificity, and F1-score are just a few of the performance metrics that are calculated and analyzed through extensive studies. The model’s detection time during the processing of medical data is also analyzed using MBT. To illustrate how effective the proposed model is, its effectiveness has been measured against a number of contemporary deep learning models. Additionally, the models are verified using real-time statistics collected in an IoT-Fog-BAN scenario. According to the findings, the recommended model outperformed the other models by a wide margin and fits its strong point in the IoT-Fog-BAN Environment for cardiology diagnosis.

Future extensions to the suggested model’s scope will enable cost-effective ways to preserve the high QoS (Quality of Services) features. Since the Fog gateways’ model training demands more computational power, federated decentralized training patterns with less hardware resources can take the place of traditional training methods. These patterns can then be expanded to other domains, including automation, smart homes, and agriculture.

## Data availability statement

The raw data supporting the conclusions of this article will be made available by the authors, without undue reservation.

## Ethics statement

Ethical approval was not required for the studies involving humans because research that involves anonymous surveys with no personally identifiable information may be considered low-risk and not require formal ethical approval. The studies were conducted in accordance with the local legislation and institutional requirements. Written informed consent for participation was not required from the participants or the participants’ legal guardians/next of kin in accordance with the national legislation and institutional requirements because if the research poses minimal risk to participants, the IRB might waive the requirement for written informed consent. Minimal risk studies are those in which the probability and magnitude of harm or discomfort anticipated are not greater than those ordinarily encountered in daily life or during the performance of routine physical or psychological examinations or tests.

## Author contributions

RA: Methodology, Writing – original draft. PE: Conceptualization, Writing – original draft.
